# Determinants of body image disturbance and disordered eating behaviors among self-identified LGBTQ individuals

**DOI:** 10.1186/s40337-023-00810-2

**Published:** 2023-06-02

**Authors:** Nour Kalash, Hana Harb, Nadine Zeeni, Myriam El Khoury, Lama Mattar

**Affiliations:** 1 Nutrition Program, Department of Natural Sciences, School of Arts and Sciences, Lebanese American University, P.O. Box 13-5053, Chouran, Beirut, 1102 2801 Lebanon; 2 Psychology Program, Department of Social Sciences, School of Arts and Sciences, Lebanese American University, Beirut, Lebanon

**Keywords:** Disordered eating behaviors, Body image disturbance, Sexual minorities, Gender identity, And sexual orientation

## Abstract

It has been previously established that sexual minorities are more likely to suffer from mental health illnesses due to experiencing unique stressors such as fear, anxiety, stigma, harassment, and prejudice. Results revealed two noteworthy mental health disorders reported by lesbian, gay, bisexual, transgender, and queer (LGBTQ) individuals, disordered eating behaviors and disturbed body image. Yet, a-priori studies revealed inconsistent results with regards to body image concerns and eating disorder symptoms and attitudes among sexual minorities. Therefore, this cross-sectional study aimed at investigating the occurrence of disordered eating behaviors (DEB) and body image disturbance (BID) among sexual minorities in Lebanon. Additionally, the correlation between several determinants of DEB and BID including fear of negative evaluation, generalized anxiety, social support, and harassment levels have been examined. Generally, the present study revealed that LGBTQ population scored higher than cisgender and heterosexual individuals for the mean and global scores of the EDE-Q6.0 and BAS-2. Only generalized anxiety and fear of negative evaluation scales were significantly associated with DEB and BID among different sexual orientation and gender identity individuals. Therefore, it is very important that health professionals working with such vulnerable populations meticulously assess for disordered eating behaviors and body image disturbance for better communication and management.

**Supplementary Information:**

The online version contains supplementary material available at 10.1186/s40337-023-00810-2.

## Abstract (plain language summary)

Sexual minorities are individuals who are more likely to experience mental health illnesses due to their increased experiences of social stresses, such as stigma, discrimination, prejudice, and fear of rejection. Two of the common mental health illnesses lesbian, gay, bisexual, transgender, and queer individuals develop are eating disorders and disturbed body image. Eating disorders and body image concerns are serious mental health illnesses with the likelihood of progressing into life-threatening conditions. Behavioral characteristics of eating disorders can be seen as binge eating episodes, extreme dieting, and inappropriate compensatory behaviors such as fasting, self-induced vomiting, excessive exercise, laxative or diuretic misuse, and or misuse of other “weight loss/diet” pills. Disordered eating patterns and body image concerns are multifactorial and complex, involving a wide range of psychological, sociocultural, and biological determinants. The current study is one of the few observational studies that tackle sexual minority health related issues, specifically investigate the presence and determinants of body image disturbances and disordered eating behaviors in gay, lesbian, bisexual, transgender, and queer individuals compared to heterosexual and/or cis-gender population in Lebanon. The study findings suggest a potential higher risk of disorder eating behaviors and body image disturbances among sexual and gender minorities.

**Supplementary Information:**

The online version contains supplementary material available at 10.1186/s40337-023-00810-2.

## Background

Body image disturbance and disordered eating patterns have been reported among cis-gender and heterosexual individuals especially women [23, 59]. However, recent studies have been suggesting that sexual minorities may be a particular group at risk for the development of disordered eating behaviors (DEB) and body image dissatisfaction (BD) [38, 44]. Body image disturbance (BID) or negative body image is a concept referring to high body image dissatisfaction (BD), weight dissatisfaction, negative perception of body, appearance evaluation and high drive for thinness [12]. Sexual minorities (SM) are a group whose gender identity, sexual orientation and/or practices differ from what is commonly known as the “norm” in the surrounding society [49]. Typically, sexual minorities include lesbian, gay, bisexual, and transgender individuals. Despite the thriving body of research that has examined sexual-orientation based differences, it is still unclear whether sexual minorities experience BID and DEB as frequently as their heterosexual counterparts.

To date, many studies are still investigating the relationship between BID and DEB in SM. Results from previous research studies on gender and sexual minorities propose a distinctive correlation between gender identity and sexual orientation with disordered eating patterns and attitudes [17]. Contemporaneously, sexual orientation and gender identity may interrelate with eating patterns and weight concerns. Himmelstein et al., [23] highlighted that sexual and gender minorities have a stronger possibility than heterosexuals to engage in disordered eating behaviors or harmful weight-control approaches. For example, some work showed there were no differences in sexual orientation between women’s [39] or men’s [20] body dissatisfaction levels. On the other hand, certain studies have found differences linked to sexual orientation; notably a higher risk of BID in heterosexual females compared to Lesbian females [3, 47] as well in heterosexual men compared to gay men [45, 58]. Strong et al. [52], also concluded that body image concerns and eating disorder symptoms were similar for heterosexual females, gay males, and lesbians, but were different for heterosexual males. However, the majority of studies have shown that sexual minority men are at higher risk of body image dissatisfaction than their heterosexual counterparts [13, 19, 21]. With regards to DEB, Himmelstein et al., [23] highlighted that sexual and gender minorities have a stronger tendency to engage in DEB or harmful weight-control approaches compared to heterosexuals. A very recent review article noted a higher prevalence of Eating Disorders (ED) and DEB patterns among gay, bisexual, and transgender adults and adolescents [44]. Further stressing on new studies, disordered eating patterns and body image are being considered major concerns among sexual and gender minorities [42]. However, it can be noted from previous studies that much of the research done on eating disorders has been examined in cis-gender and heterosexual populations despite the documentation of high rates of disordered eating among SM [23].

The contradictory results regarding the differences between heterosexuals and SM may stem from how individuals respond differently to stressful events. LGBTQ individuals endure unique stressors in their everyday lives that may explain their BID and DEB levels. According to the “Minority Stress” theory [34], LGBTQ-identified individuals who are categorized under sexual and gender minorities are more exposed to stress, stigma, and prejudice compared to their heterosexual counterparts. Additionally, the above theory claims that discrimination and harassment based on sexual orientation results in less social support from family, more social anxiety and more general negative perception of one’s self, all of which are linked to BID and DEB [28]. It has been previously mentioned that minority stress may contribute to elevated levels of attitudinal disordered eating patterns among lesbian women, gay men, bisexual men and women, and transgender women [9, 28, 34, 42] b). According to Meyer [34], the impact of minority stress on mental health can be diluted through social support. In fact, Burnette et al. [7] showed that social support was positively correlated with body appreciation through resilience and self-esteem in lesbian, bisexual, and queer women.

Additionally, in some countries such as Lebanon, same-sex couples are considered illegal and “contrary to nature” facing daily risks of prosecution. LGBTQ individuals are thus marginalized ensuring in fear of living the country or displaying their identity/sexual orientation [27]. Subsequently, such experiences increase their levels of anxiety, fear, stigmatization, prejudice, discrimination and rejection triggering numerous mental and psychological issues [36, 27]. Based on the above considerations, the aim of the present study is to investigate the presence and determinants of BID and DEB in gay, lesbian, bisexual, transgender and queer individuals compared to a heterosexual and/or cis-gender population in Lebanon, and to highlight the role of psychological factors such as anxiety, fear of negative evaluation, social support, harassment and discrimination in this relationship.

## Methods

### Study design and ethical considerations

This is an observational, cross-sectional and population-based study, carried out on interested individuals of different gender identities and sexual orientations. The participants were contacted through social media accounts such as Instagram LLC., Twitter Inc., and Facebook Inc., in addition to referrals, and non-governmental organizations focused on human rights. The participants were clearly given the option to withdraw their surveys from the study with both anonymity and confidentiality being assured. See Additional file 1: Appendix A.

The outcomes of this study were measured through an online survey administered on google docs (Google LLC.) displayed in both languages English and Arabic. The scales were translated from English to Arabic and back translated to English, by a professional translator. Study participants were given the option to choose the language of instruction and were able to access an educational brochure after submitting the survey. The brochure entitled “Your Guide to a Healthy Eating and Wellbeing” was developed by the researchers as an appreciation for completing the study survey. See Additional file 1: Appendix I.

The above study was approved by the Institutional Review Board Office at the Lebanese American University “LAU.SAS.LM4.10/Sep/2020”, which is constituted in accordance with the US Code of Federal Regular (45 CFR46. 107, 21CFR, 56.107), and Good Clinical Practice ICH.

### Study participants

Inclusion criteria included self-identified lesbian, gay, bisexual, transgender, queer and heterosexual men and women above 16 years old and residing in Lebanon from October 2020 till April 2021. Three participants were excluded from the study analysis for they reported “Other” under gender identity and sexual orientation, thus not falling under the LGBTQ categories.

### Outcome measures

#### Sociodemographic and sexual identity

Participants were asked about their sociodemographic and educational statuses. They were also asked to identify their sex at birth (male, female or intersex), gender identity (woman/cis-woman, man/cis-man, trans-woman, trans-man, gender queer or “other”) and sexual orientation (straight/heterosexual, lesbian, gay, bisexual, asexual, queer or “other”).

#### Eating disorder examination questionnaire (EDE-Q-6.0)

To assess eating behaviors, the Eating Disorder Examination Questionnaire (EDE-Q-6.0) was deemed most fit for this study. It is a self-report questionnaire that evaluates disordered eating behaviors and eating disorder attitudes over the past 28 days [16]. The assessment demonstrates four subscale scores each over a 7-point order response: Restraint Eating (5 items), Eating Concern (5 items), Shape Concern (8 items), and Weight Concern (5 items). The Global score is measured as the average of these subscales with higher scores indicative of eating- related behaviors or concerns. To determine proneness or higher risk of disordered eating behaviors, a cut-off of 4 was used [33]. The EDE-Q global score has demonstrated high internal consistency (Cronbach’s α = 0.94) for the global score. See Additional file 1: Appendix C.

#### Body appreciation scale (BAS-2)

Body image disturbance was assessed using the second version of the body appreciation scale (BAS-2) [56]. Results of lower body appreciation is an indicative of higher body image dissatisfaction and hence body image disturbance [1, 4, 12]. It includes 10 items that are graded on a 5-point Likert scale ranging from *never* (accounting to a score of 1) to *always* (accounting to a score of 5). BAS-2 is scored by summing up the item responses with lower scores indicative of lower body appreciation thus higher body image dissatisfaction. This scale was validated among western and non-western population [5]. Additionally, the BAS-2 is considered as a validated tool among sexual minorities for assessing its correlation with other aspects of positive body image, inclusive of body image flexibility and functionality appreciation [53]. Scores on the BAS-2 have demonstrated excellent internal consistency (Cronbach’s α = 0.96). See Additional file 1: Appendix D.

#### Generalized anxiety disorder (GAD-2)

The Generalized Anxiety Disorder 2-item (GAD-2) scale was used to evaluate participants’ anxiety level. It is considered as a very brief and simple initial screening tool for frequency of general anxiety disorder over the preceding two-week period. Responses are graded on a four-point Likert scale ranging from 0 (not at all) to 3 (nearly every day). A score of 3 points is the selected cut-off for pointing out possible cases that are in need for further evaluation [25]. Internal consistency as demonstrated by Cronbach’s alpha (Cronbach’s α = 0.91). See Additional file 1: Appendix E.

#### Brief fear of negative evaluation (BFNE)

The Brief Fear of Negative Evaluation was used to further assess social anxiety among the LGBTQ community. The BFNE is a 12- item scale based on a five-point Likert scale ranging from 1 (not at all characteristic of me) to 5 (extremely characteristic of me); the higher the score, the greater the fear of negative evaluation and perception of others towards oneself. Several studies before have used the BFNE scale to assess the level of fear of negative evaluation and social anxiety among lesbian, gay, bisexual, and transgender individuals [32, 50]. Additionally, scores on BFNE has demonstrated high internal consistency (Cronbach’s α = 0.86). See Additional file 1: Appendix F.

#### Heterosexist, harassment, rejection, and discrimination scale (HHRDS)

The 14-item heterosexist harassment, rejection, and discrimination scale (HHRDS) was used to measure the frequency that the LGBTQ minorities report experiences of heterosexist harassment, rejection, and discrimination over the past year. Responses are rated on a 6-point Likert scale that ranges from 1 (the event never happened to you) to 6 (the event happened almost all of the time). High total scores indicated more discriminatory experiences. Moreover, previous research studies have used the HHRDS in evaluating harassment and discrimination events among lesbian, gay, and transgender individuals [51, 54],Tabaac et al., 2017). Moreover, HHRDS scale exhibited excellent internal consistency (Cronbach’s α = 0.94). See Additional file 1: Appendix G.

#### Multidimensional scale of perceived social support (MSPSS)

The Multidimensional scale of perceived social support (MSPSS) was included to evaluate the subjective apprehension of social support adequacy in three specific aspects: family, friends, and significant others (Zimet et al., 1998). The MSPSS consists of 12-items rated from 1 (strongly disagree) to 5 (strongly agree), with total scores representing the sum of responses. Moreover, the higher the scores the greater the perceived social support status. MSPSS scores exhibited high internal consistency (Cronbach’s α = 0.92). See Additional file 1: Appendix H.

### Statistical analysis

Statistical analysis was performed using SPSS version 25. Descriptive analyses are represented as mean and standard deviation for continuous variables and percentages for categorical. Cross tabulation tables were done to further review the characteristics of our participants within subgroups. Descriptive statistics were also performed to calculate the mean score of scales and their subscales. Sample t-test was conducted to test for difference in the mean score and the global score of the EDE-Q-6.0 subscales in addition to the mean score of the BAS-2 between LGBQ and heterosexuals. Additionally, ANOVA tests were conducted to test the difference in the mean score of the scales between the gender identity and sexual orientation subgroups. A General linear model was performed to test the relation between the global EDE-Q6.0 score and BAS-2 with both gender identity and sexual orientation. Pearson correlations were conducted for each determinant variable (body appreciation, anxiety, fear of negative evaluation, social support, and harassment scales) to check for multicollinearity and strength of association with controlling for age and BMI. Multiple linear regression was performed to test the association of predicted determinants of both disordered eating behaviors and body image disturbance. The included scales were generalized anxiety disorder, fear of negative evaluation, perceived social support status, and harassment/rejection experiences. In addition to BAS-2 as a determinant for disordered eating behaviors. Then, the strength of association was analyzed using correlation coefficients between independent variables and the outcome. Significance was considered at *p* < *0.05*. Cronbach's alpha was calculated for each of the scales used to assess their internal consistency.

## Results

### Characteristics of participants

The number of participants included for the final analysis was *358* of which the number of gender identity and sexual orientation groups are presented in Table 1. Participants had a mean age of 24 years with a mean BMI of 24.17 kg/m^2^. As for the area of residence, the majority of the participants lived in Beirut and Mount Lebanon (51.5% and 36.3%, respectively) and the rest were distributed among Lebanese governorates.

**Table 1:** Cross tabulation of participants’ gender identity and sexual orientation along with age and BMI in mean and standard deviation

		Frequency	Age (Mean ± SD)	BMI (Mean ± SD)
Gender identity	Cis-Man	134 (37.4%)	26 ± 6	25.7 ± 5
	Cis-Woman	187 (52.2%)	23 ± 4	22.9 ± 4.2
	Trans-man	7 (2%)	20 ± 3	27.5 ± 4.7
	Trans-woman	4 (1.1%)	22 ± 4	24.5 ± 2.3
	Queer	26 (7.2%)	23 ± 3	24.6 ± 4.7
Sexual orientation	Heterosexual Men	52 (14.8%)	27 ± 6	25.6 ± 3.6
	Heterosexual Women	102 (29.8%)	24 ± 4	22.5 ± 3.8
	Gay	76 (21.1%)	25 ± 6	25.5 ± 5.4
	Lesbian	24 (6.6%)	23 ± 4	23.5 ± 5.2
	Bisexual	62 (17.2%)	21 ± 3	23.7 ± 5.1
	Queer	25 (6.9%)	22 ± 4	25.1 ± 3.6
	Asexual	13 (3.6%)	27 ± 7	23.16 ± 4.35
Total			24 ± 5	24.17 ± 4.73

Age: in years; BMI: body mass index (Kg/m^2^); SD: standard deviation

### Body image disturbance and disordered eating behaviors among sexual minorities participants

Table 2 shows that lesbian, gay, bisexual, queer, and asexual (LGBQA) ranked significantly lower on the BAS-2 scale compared to heterosexual participants. A significant difference in the mean scores between LGBQA and heterosexual participants in both shape concern (*p* < 0.001) and weight concern (*p* < 0.001) subscales was detected. In both subscales LGBQA scored higher than heterosexual participants, indicating higher concerns among this group. With regards to gender identity, there was a significant difference in the mean scores of BAS-2 between the groups (F = 9.029; *p* < 0.001) where Trans and Queer gender (TQ) individuals reported lower BAS compared to cis-men and cis-women and higher weight and shape concerns compared to both cis- men and women (Table 3).

**Table 2:** Mean scores and standard deviation for the scales between Heterosexual and LGBQA

	Heterosexual		LGBQA		t (2-tailed sig)
	N	Mean ± SD	N	Mean ± SD	
BAS-2	159	3.74 ± 1.08	200	3.23 ± 1.02	4.59**
EDE-Q 6.0 Global Score	159	1.52 ± 1.07	200	1.99 ± 1.35	− 3.67**
Restraint Eating	159	1.47 ± 1.58	200	1.79 ± 1.79	− 1.76
Eating Concern	159	0.81 ± 0.96	200	1.36 ± 1.55	− 4.11**
Shape Concern	159	2.04 ± 1.23	200	2.51 ± 1.39	− 3.37**
Weight Concern	159	1.76 ± 1.24	200	2.30 ± 1.41	− 3.85**
GAD-2	159	1.71 ± 0.94	200	2.04 ± 0.951	− 3.28**
BFNE	159	32.6 ± 9.69	200	36.1 ± 10.66	− 5.57**
MSPSS	159	5.19 ± 1.42	200	4.72 ± 1.42	3.09**
HHRDS		N/A	200	2.48 ± 1.24	N/A

**Statistical Significance is set at the 0.01 level (2-tailed); BAS., Body Appreciation Scale; EDE-Q 6.0, Eating Disorder Examination Questionnaire; LGBQA., Lesbian, Gay, Bisexual, Queer, Asexual; GAD., Generalized Anxiety Disorder; BFNE., Brief Fear of Negative Evaluation; HHRDS., Heterosexist, Harassment, Discrimination and Rejection; MSPSS., Multidimensional Scale of Perceived Social Support; N/A, not applicable

**Table 3:** Scales Mean Scores and Standard Deviation of the scales among Gender Identity Groups

	Gender Identity					
	Cis-Man		Cis-Woman		TQ	
	N	Mean ± SD	N	Mean ± SD	N	Mean ± SD
GAD-2	134	1.73 ± 0.96	187	2.04 ± 0.951	37	2.18 ± 0.90
BFNE	134	33.76 ± 9.82	187	34.71 ± 10.63	37	36.32 ± 10.60
MSPSS	134	4.90 ± 1.36	187	4.99 ± 1.50	37	4.71 ± 1.38
HHRDS	82	2.35 ± 1.09	85	2.38 ± 1.36	33	3.08 ± 1.09

GAD, Generalized Anxiety Disorder; BFNE, Brief Fear of Negative Evaluation; HHRDS, Heterosexist, Harassment, Discrimination and Rejection; MSPSS, Multidimensional Scale of Perceived Social Support; TQ, transgender and queer-gender

With regards to DEB, the mean scores for all of the four subscales and global score for the EDEQ-6.0 were higher in the LGBQA group than heterosexual individuals (Table 2). Mean score above 4 was not seen in any of the two groups for all of the subscales and the global score. However, there was a significant difference between the two groups for the eating (*p* < 0.001), shape (*p* < 0.001), and weight concern (*p* < 0.001) subscales of the EDE-Q. Moreover, the difference was also present for the global score between the two groups (*p* < 0.001), thus, LGBQA individuals were more prone to DEB.

Mean scores for the GAD-2, BFNE, MSPSS, and HHRDS for the groups: heterosexual/straight, transgender/gender queer and LGBQA individuals are listed in Tables 2 and 3. LGBQA individuals scored higher on the GAD-2 and BFNE while heterosexual participants scored higher on the MSPSS, seen in Table 2. Additionally, transgender and gender queer participants scored higher than their cis-counterparts on GAD-2 and BFNE, seen in Table 3. With regards to social support, heterosexuals and cis-genders scored significantly higher compared to LGBQA and TQ indicative of higher social support.

### Association between anxiety, fear of negative evaluation, social support, harassment and discrimination with body image

After correcting for age, BMI, level of education, exercise level and area of residence, a significant regression equation was found (*p* < 0.01), and 31% of the variance in the BAS-2 is described by the determinants mentioned above (R^2^ = 0.307). BAS increased as social support increased, and decreased as BFNE, GAD and HHRDS increased. GAD (*p* < 0.05), Fear of negative evaluation (*p* < 0.001) and social support (*p* < 0.05) were significant predictors of BAS. Additionally, results indicated that MSPSS is a more significant predictor of BAS than other factors through higher standardized coefficient of 0.142. It is crucial to note that GAD became significant after adjusting for area of residence.

Pearson correlation test was conducted to examine the strength of the relationship between GAD-2, BFNE, MSPSS, HHRDS, and BAS-2 (Table 4). Results suggest a weak significant inverse correlation between GAD-2 and BAS-2 (r = − 0.374; *p* < 0.01), likewise there was a weak negative correlation between HHRDS and BAS-2, but it was significant (r = − 0.147; *p* < 0.05). Furthermore, there was a moderate inverse correlation between BFNE and BAS and this correlation was strongly significant (r = − 0.509; *p* < 0.01). This indicates that as GAD- 2, BFNE and HHRDS increase, body appreciation decreases. On the other hand, MSPSS was positively correlated to BAS-2 (r = 0.301; *p* < 0.01) indicative of a protective effect in which body appreciation rises whenever the social support increases.

**Table 4:** Pearson Correlation between BAS-2 and determinant variables

	BAS-2	GAD-2	BFNE-2	MSPSS	HHRDS
BAS-2	1	− 0.374**			
GAD-2	− 0.374**	1			
BFNE-2	− 0.509**	0.448**	1		
MSPSS	0.301**	− 0.171**	− 0.216**	1	
HHRDS	− 0.147	0.258**	0.060	− 0.265**	1

BAS., Body Appreciation Scale; GAD-2., Generalized Anxiety Disorder-2; BFNE., Brief Fear of Negative Evaluation; HHRDS., Heterosexist, Harassment, Discrimination and Rejection; MSPSS., Multidimensional Scale of Perceived Social Support; **Statistical significance is set at the 0.01 level (2-tailed)

### Association between body appreciation, anxiety, fear of negative evaluation, social support, harassment and discrimination with disordered eating behaviors

After calculating the mean and standard deviation scores for the above questionnaires among both gender identity and sexual orientation groups we conducted regression analysis. The analysis indicated there was a strong significant relationship between all of the 5 determinant scales mentioned above and the EDE-Q among both gender identity and sexual orientation groups (*p* = 0.00 < 0.01). Thirty-five percent of the variance in the EDE-Q is accounted by the determinants mentioned above (R^2^ = 0.349). Therefore, we reject the null hypothesis that the determinants are not related to the dependent variable (global EDE-Q score). Diving into each of the determinants, only the body appreciation (*p* < 0.001), generalized anxiety (*p* = 0.025), and fear of negative evaluation (*p* = 0.008) were significant. The results indicated a negative relationship between body appreciation and disordered eating behaviors, as the BAS-2 score increases by one unit, the global EDE-Q score decreases by 0.528.

On the other hand, the generalized anxiety and fear of negative evaluation had a positive relationship with disordered eating behaviors. Thus, when the GAD score increases by one unit, the global EDE-Q score increases by 0.205 and when the BFNE score increases by one unit, the global EDE-Q score increases by 0.023.

Finally, the strength of association between the global EDE-Q score and each of the determinants is displayed in Table 5 through Pearson Correlation. The results indicated a negatively significant correlation between global EDE-Q score with the BAS-2 score (r = 0.555 = medium strength association) and with the MSPSS (r = 0.203 = weak strength association). Moreover, there was a positively significant correlation between the global EDE-Q score with the GAS-2 (r = 0.344 = medium strength association) and the BFNE scores (r = 0.424 = medium strength association).

**Table 5:** Pearson Correlation between the global score EDE Q6.0 and determinant variables

	Global EDE-Q6.0 (Four subscales)	BAS-2	GAD-2	BFNE-2	MSPSS	HHRDS
Global EDE-Q6.0 (Four subscales)	1					
BAS-2	− 0.555**	1				
GAD-2	0.344**	− 0.374**	1			
BFNE-2	0.424**	− 0.509**	0.448**	1		
MSPSS	− 2.03**	0.301**	− 0.171**	− 0.216**	1	
HHRDS	0.124	− 0.147**	0.258**	0.060	− 0.265**	1

EDE-Q., Eating Disorder Examination Questionnaire; BAS-2., Body Appreciation Scale-2; GAD-2., Generalized Anxiety Disorder-2; BFNE., Brief Fear of Negative Evaluation; HHRDS., Heterosexist, Harassment, Discrimination and Rejection; MSPSS., Multidimensional Scale of Perceived Social Support **Correlation is significant at the 0.01 level (2-tailed)

## Discussion

Primary outcomes revealed predominance of BID and DEB among sexual minorities compared to heterosexual participants. Interpretation of secondary outcomes validated that low levels of social support and high levels of anxiety and fear of negative evaluation were found to be determinants of both BID and DEB among the identified lesbian, gay, bisexual, transgender, queer, and sexual (LGBTQA) community. Another determinant for BID were the high levels of harassment, discrimination, and prejudice. As for social support, LGBTQA-identified individuals had lower levels compared to heterosexual and cis-gender participants.

Further explaining our results, the predominance of BID was evident by the lower body appreciation and higher weight and shape concerns among LGBQA compared to heterosexual participants. As for DEB, the predominance was seen among LGBQA compared to heterosexual participants through higher scores of all four subscales and the global score of the eating disorder examination questionnaire. Despite not having a significant difference for the mean score of the restrain concern, LGBQA had higher concerns compared to heterosexual participants implying, in the same line of previous literature, a higher risk for disordered eating patterns and attitudes among sexual and gender minorities [6, 23, 44]. According to a-priori studies on mental health disparities among LGBTQ populations, there are several factors that might play a role in either endorsing or hindering the development of disordered eating symptoms and body image disturbance. One leading framework that can explain the greater threat of DEB and BD to the LGBTQ community is the minority stress theory [35]. Sexual minorities such as LGBTQ individuals endure many traumatic experiences during their lives that are associated with discrimination, harassment, fear of negative evaluation, anxiety, stigma, and lack of social support [35]. Such experiences can create a stressful environment for sexual minorities to live in. Consistent with the minority stress theory [34] and former studies [8, 11, 24], our results prove that LGBTQA individuals experience significantly higher stressors compared to heterosexual individuals.

Diving deeper into the relationship between body image with the above-mentioned stressors, social support, generalized anxiety, fear of negative evaluation, harassment, rejection, and discrimination were significantly associated with BID in the present study. More precisely, our results indicated that as the levels of anxiety, fear, and harassment increase, body appreciation decreases and hence increasing BID. This comes in line with previous studies where fear of negative evaluation and generalized anxiety disorders are significant risk factors of BID [26, 31, 46]. Furthermore, our results indicated that body appreciation, generalized anxiety, and fear of negative evaluation scales were significantly associated with disordered eating patterns. Supporting previous literature, fear of negative evaluation and heightened levels of anxiety increase the risk of DEB among LGBTQ individuals while having higher body appreciation levels decrease that risk [6, 31, 37]. This was particularly confirmed when comparing the mean scores of the 5 determinants of DEB among heterosexual and LGBQA individuals. LGBQA participants had higher generalized anxiety and fear of negative evaluation and lower body appreciations scores than heterosexual individuals. Remembering that in almost all of our results, the weight and shape concern subscales from the EDE-Q were the highest among LGBTQ individuals, it verifies that sexual and gender minorities suffer from weight judgement and body objectification that are in return linked to the stress minority theory. This was also seen in previous literature such as Mason et al. [28] and Nagata et al. [41]. Hence, DEB may be prominent among LGBTQ individuals as a response to heightened levels of anxiety and fear, and negative perceptions of one’s self according to the stress minority theory.

As for the previously determined protective factor against DEB and BID, our results proved that as social support levels increase, the body appreciation score increases and hence, disordered eating behaviors decrease. This notion can be supported by the interpersonal theory of eating disorders (IPT-ED), another leading framework explaining the greater risk of disordered eating behaviors and body image dissatisfaction among the LGBTQ community in which lack of social support, increased perception of negative evaluation, and low self-esteem are linked to poor body image hence, higher risk of disordered eating behaviors [6]. Social support can buffer the outlined stressors mentioned above and decrease their impact on mental health [22]. In contrast, our results revealed that sexual minorities lack that buffering effect, which was evident in the lower scores on the social support scale among LGBTQA compared to heterosexual and cis-gender participants.

So far, numerous studies have shown that body image dissatisfaction is linked to DEB [2, 10, 29, 57]. In line with this, our analysis showed that BID is negatively associated with DEB, suggesting that individuals with higher BID sought disordered eating behaviors as coping strategy lessen the impact of the stressors they are exposed to. Mason et al. [29] suggested that sexual identity related experiences are associated with internalization of sociocultural norms and distorted emotion regulation which in turn results in BID and consequently, DEB.

This study in addition to previous research have been able to shed the light on the higher risk of disordered eating behaviors and body image dissatisfaction among sexual and gender minorities. However, significant gaps remain in the literature especially regarding the role of anxiety, stigma, harassment, and rejection in the relationship between sexual orientation and gender identity with DEB and BID. Thus, clinicians and researchers should focus further on the factors associated in the development of eating disorders and ED symptoms along with body image concerns among sexual minorities.

### Strengths and limitations

Similar to any other research study, the present conclusions should be interpreted in the framework of several methodological limitations. One of the major limitations of our study that limits generalizability is the inability to obtain a representative sample size of the LGBTQ community in Lebanon as sexual and gender minorities continue to live in concealment fearing prosecution and stigmatization. Another limitation is the selection bias that limits the generalizability of our results to sexual minorities of different regions since most of our participants came from Beirut and Mount Lebanon, areas that suffer from less social norms. Moreover, the questionnaires were based on “self-report”, which may subject to reporting bias. Lastly, we were not able to detect a causal relationship between our dependent and independent variable due to our cross-sectional study design.

On the other hand, our study also had certain strengths. The selected scales in this study have been carefully chosen and validated among sexual and gender minorities. More specifically, the main two scales, the BAS-2 and the EDE-Q6.0 are considered bequest instruments for body image and disordered eating behaviors respectively. The EDE-Q6.0 has been proved in numerous studies to be valid among sexual and gender minorities [42, 48].

## Conclusion

Our study findings suggest a potential higher risk of disordered eating behaviors and body image disturbance among sexual and gender minorities especially in countries where such matters remain illegal and taboo. In light of the significant stressors LGBTQ individuals endure, as they are on the road to self-discovery and disclosure, eating disorder symptoms may start to appear alarming as a result of poor body image, anxiety, fear, prejudice, and stigma according to the minority stress theory. Given the compelling groundwork on the determinants of DEB and BID among sexual and gender minorities, future research should meticulously and lengthily examine the factors involved in disordered eating behaviors and body image disturbance among the LGBTQ community in order to cultivate effective prevention strategies and enhance therapeutic interventions.

## Supplementary Information

**Additional file 1**: **Appendix A.** Informed consent. **Appendix B.** Sociodemographic, general health, and alcohol intake questionnaires. **Appendix C.** Eating disorder examination questionnaire (EDE-Q 6.0). **Appendix D.** Body appreciation scale (BAS-2). **Appendix E.** Generalized anxiety disorder (GAD-2). **Appendix F.** Brief fear of negative evaluation (BFNE). **Appendix G.** Heterosexist, harassment, rejection, and discrimination scale (HHRDS). **Appendix H.** Multidimensional scale of perceived social support (MSPSS). **Appendix I.** Your guide to a healthy eating and wellbeing.

## Data Availability

The datasets used and analyzed during the current study are available from the corresponding author on reasonable request.

## Abbreviations

LGBTQ: Lesbian, gay, bisexual, transgender, and queer
SM: Sexual minorities
ED: Eating disorders
DEB: Disordered eating behaviors
BID: Body image disturbance
EDEQ-6.0: Eating disorder examination questionnaire-version 6
BAS-2: Body appreciation scale- version 2
GAD-2: Generalized anxiety disorder- shortened version 2
BFNE: Brief fear of Negative Evaluation Scale
MSPSS: Multidimensional of Perceived Social Support Scale
HHRDS: Heterosexist Harassment, Rejection, and Discrimination Scale
