# Supplementary material for: Determinants of body image disturbance and disordered eating behaviors among self-identified LGBTQ individuals
Source: J Eat Disord. 2023 Jun 2;11:87. doi: 10.1186/s40337-023-00810-2 (PMC10239108; doi:10.1186/s40337-023-00810-2)

# **Appendices**

**Appendix A: Informed Consent**


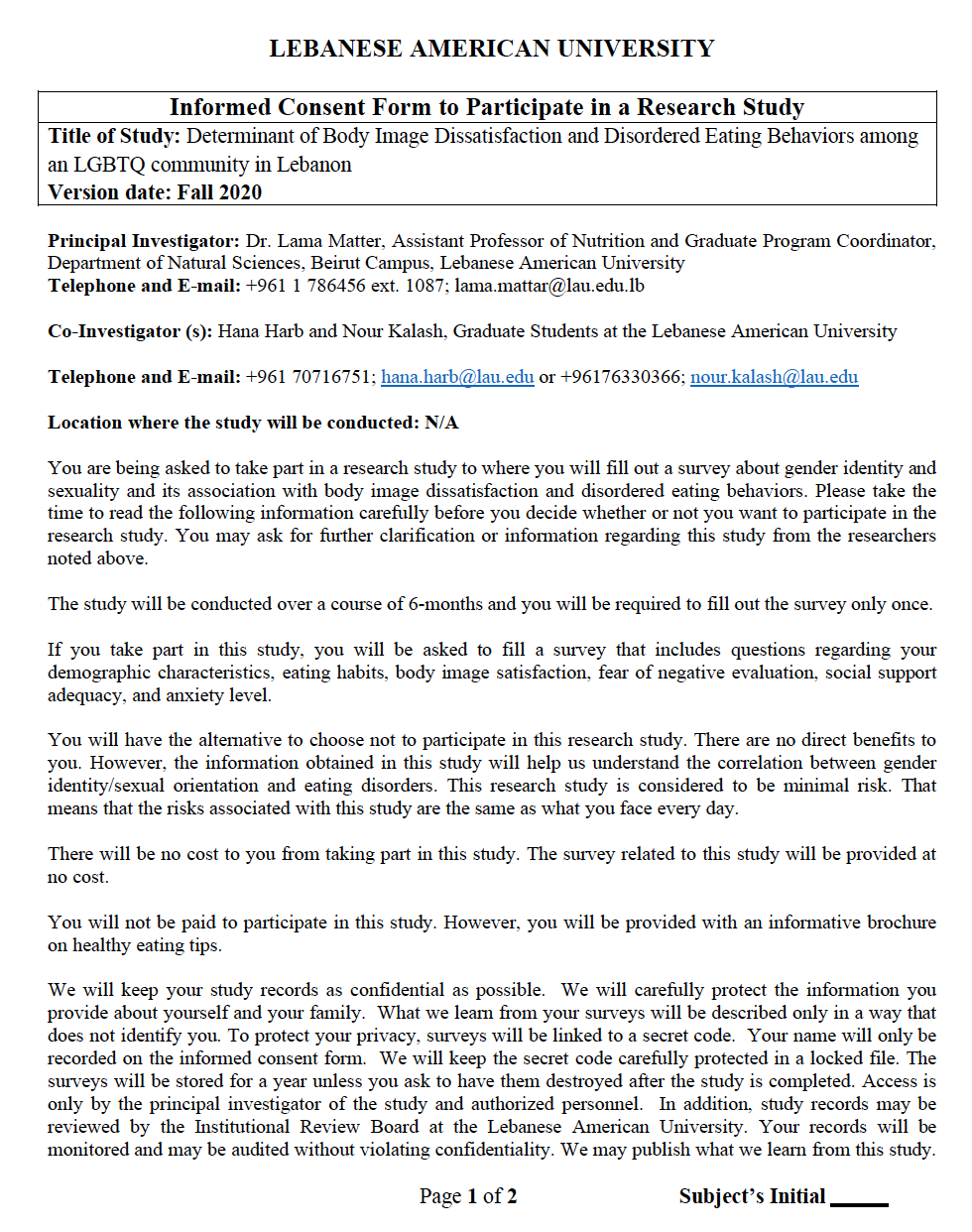


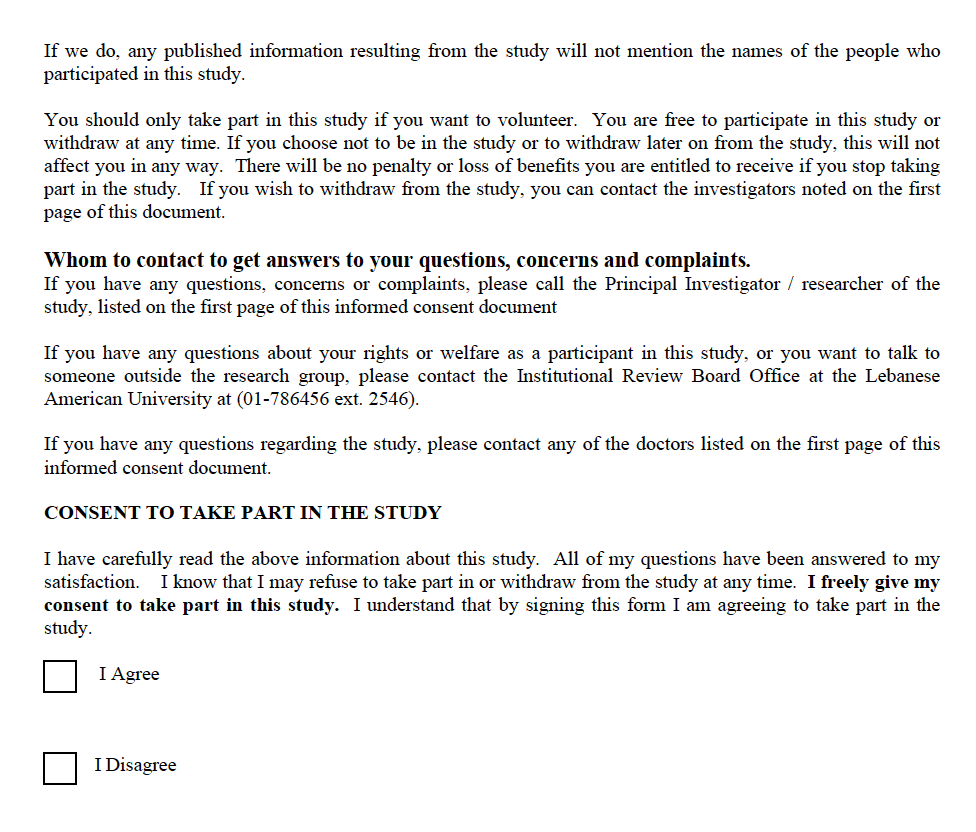


**Appendix B: Sociodemographic, General Health, and Alcohol Intake Questionnaires**


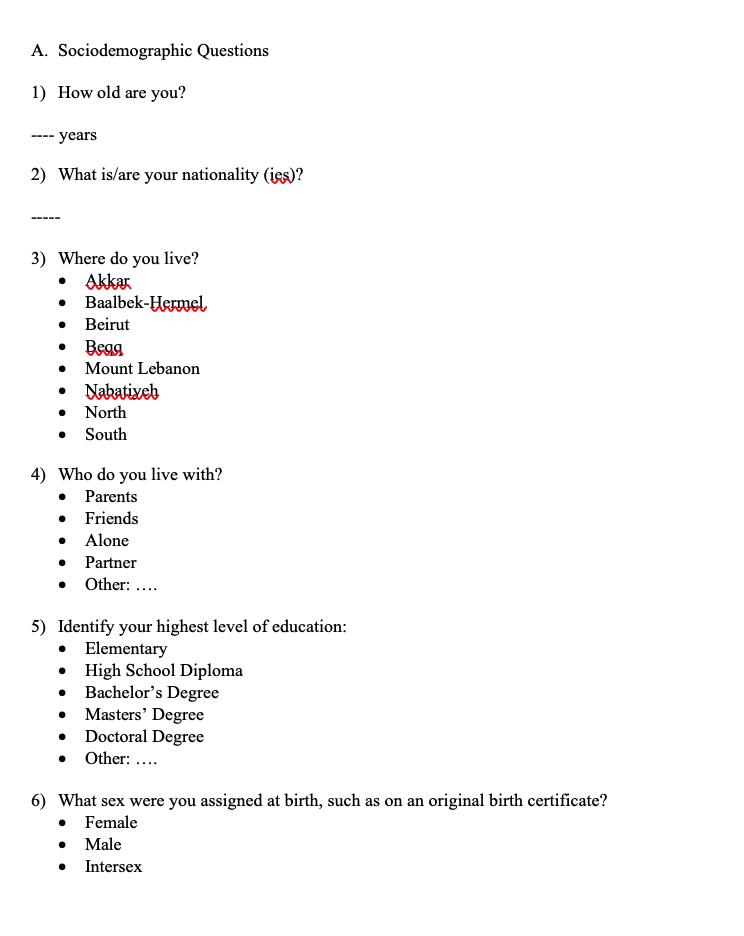


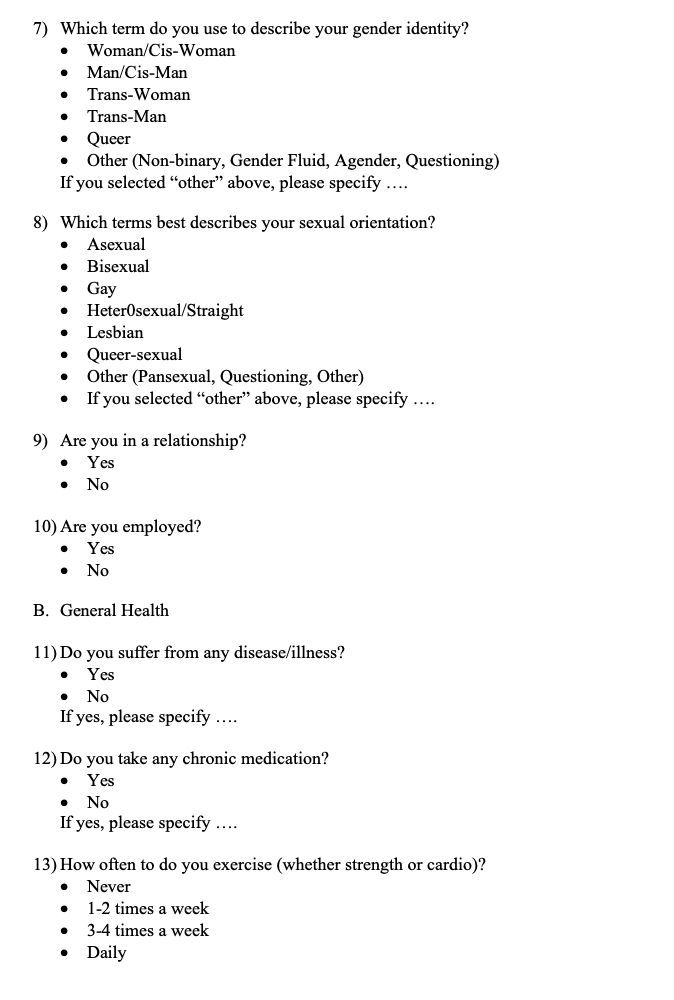


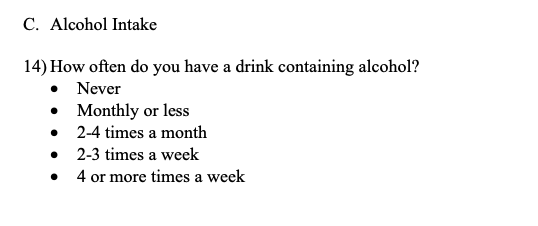


**Appendix C: Eating Disorder examination questionnaire (EDE-Q 6.0)**

**
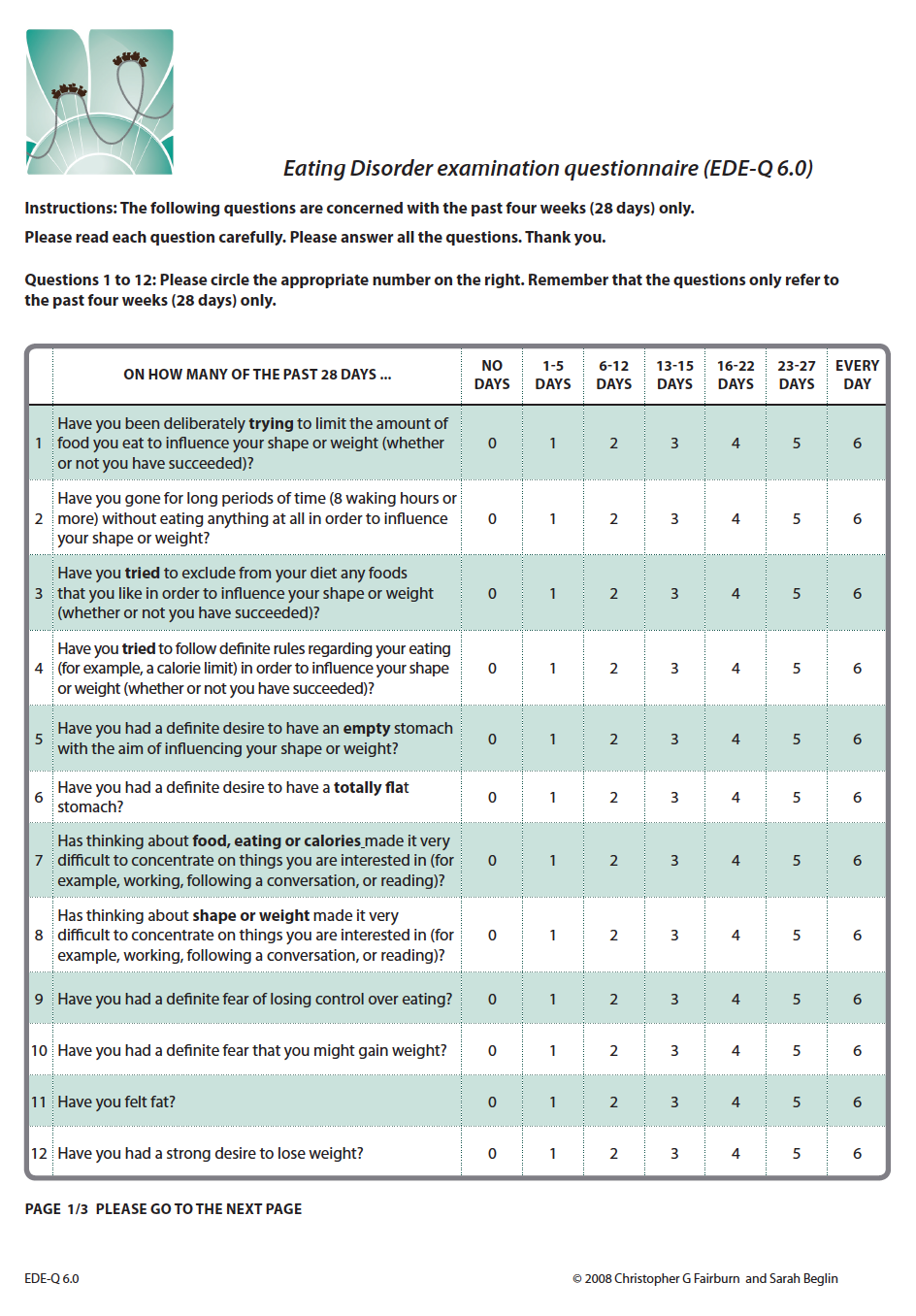
**

**
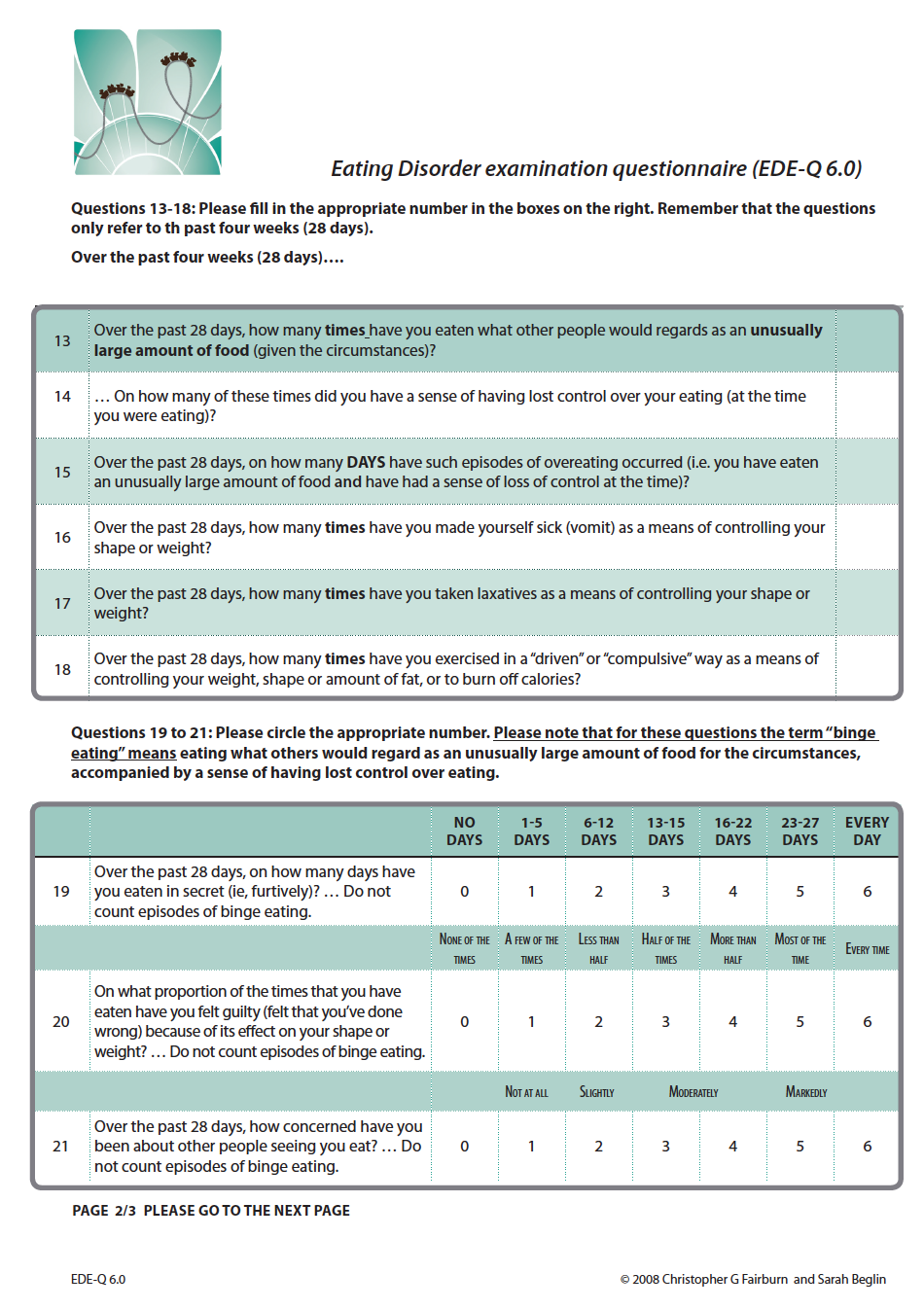
**

**
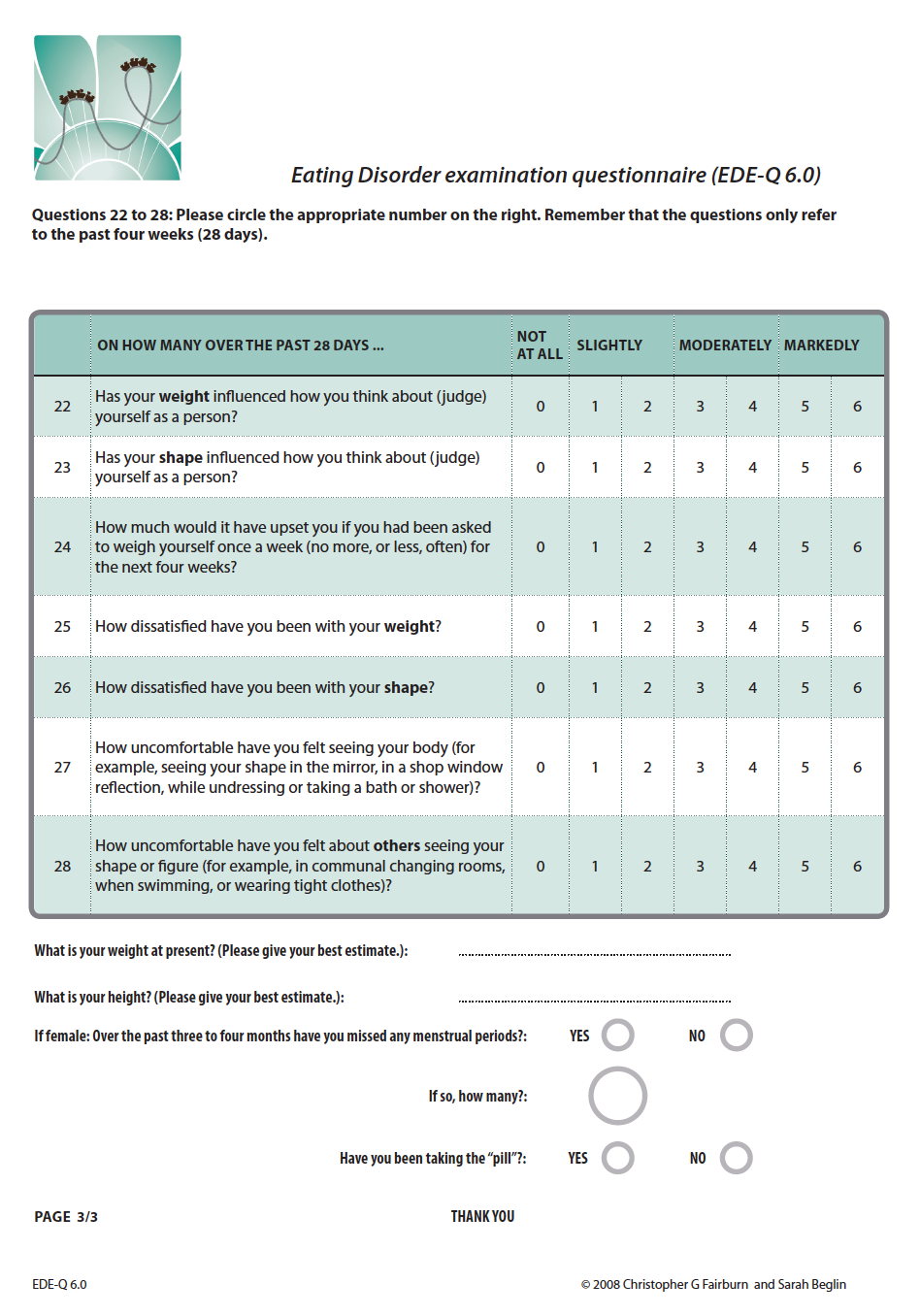
**

**Appendix D: Body Appreciation Scale (BAS-2)**

**
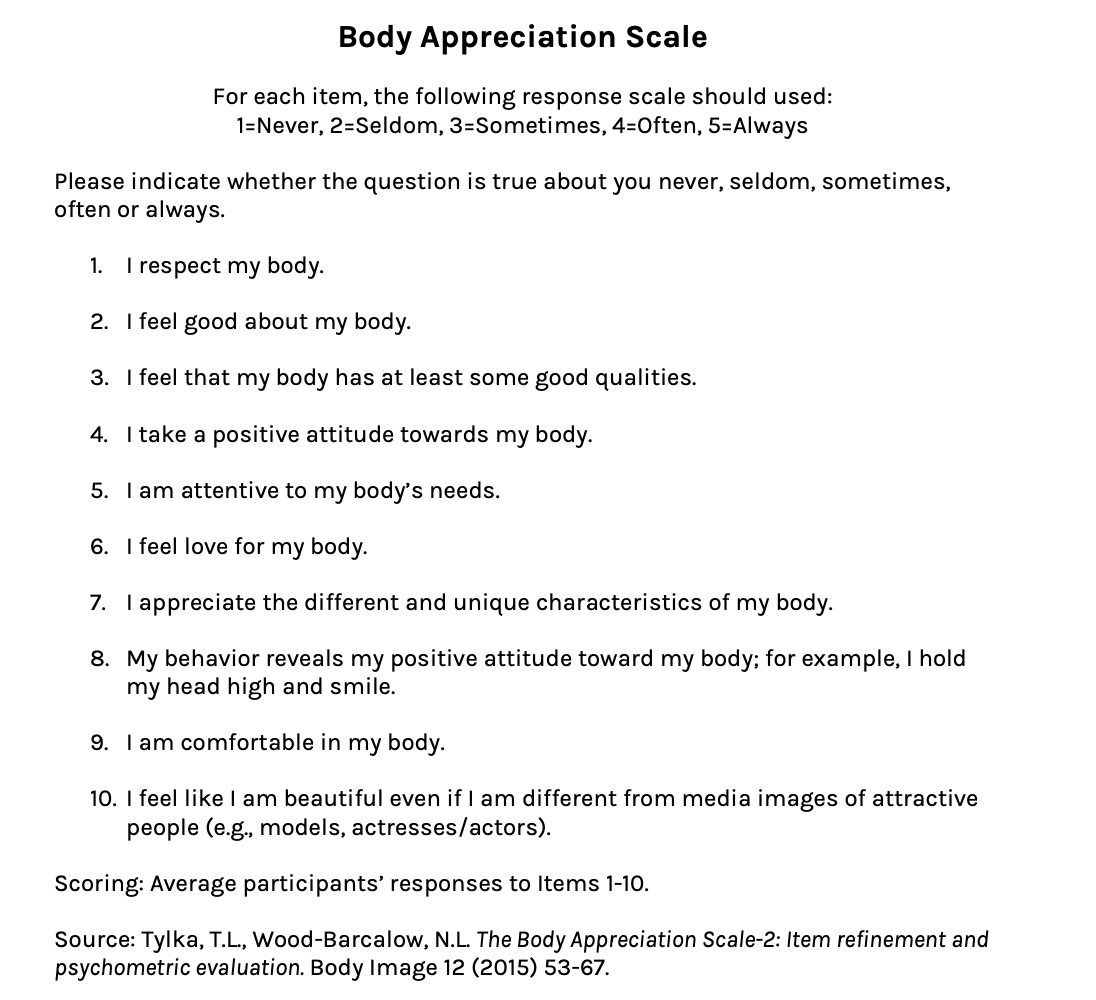
**

**Appendix E: Generalized Anxiety Disorder (GAD-2)**

**
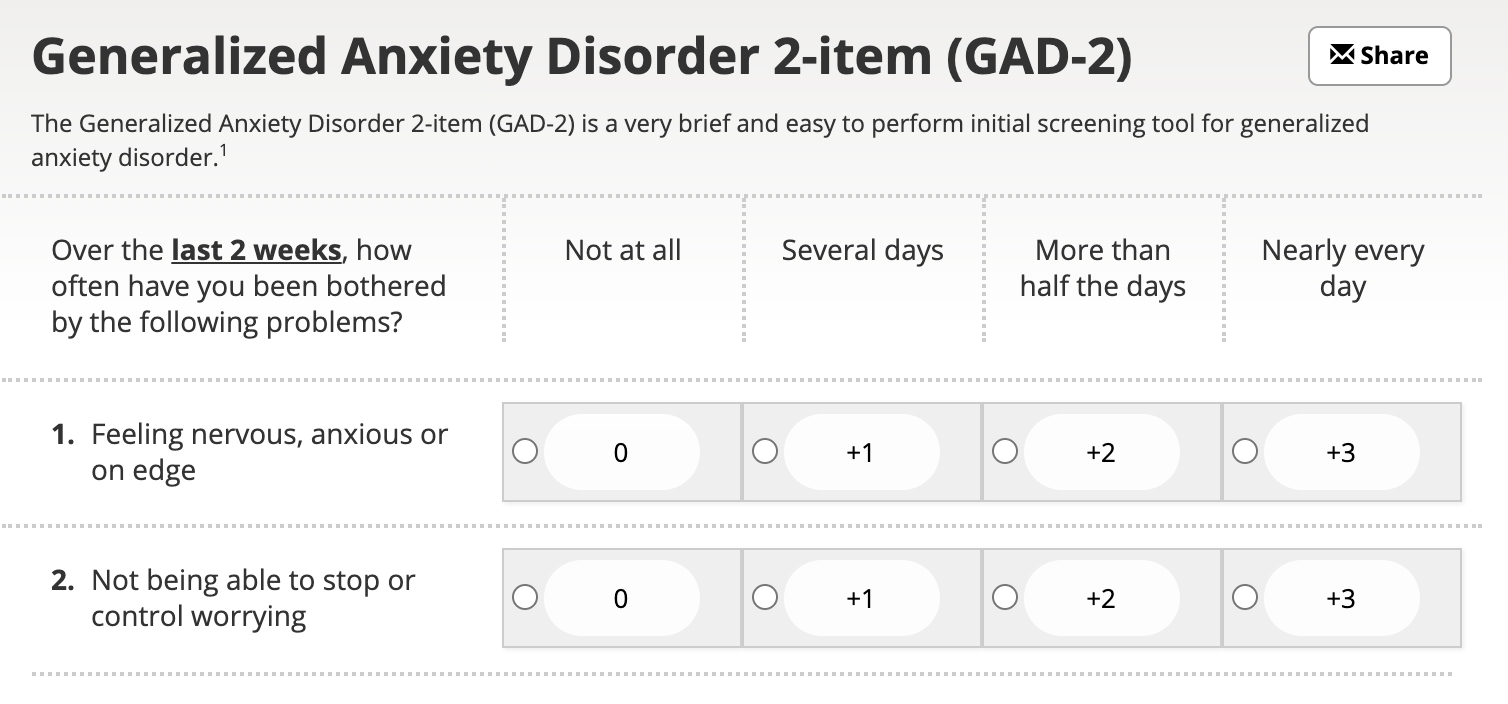
**

From: Kroenke K, Spitzer RL, Williams JB, Monahan PO, Löwe B. Anxiety disorders in primary care: prevalence, impairment, comorbidity, and detection. Ann Intern Med. 2007;146:317-25.

**Appendix F: Brief Fear of Negative Evaluation (BFNE)**

**
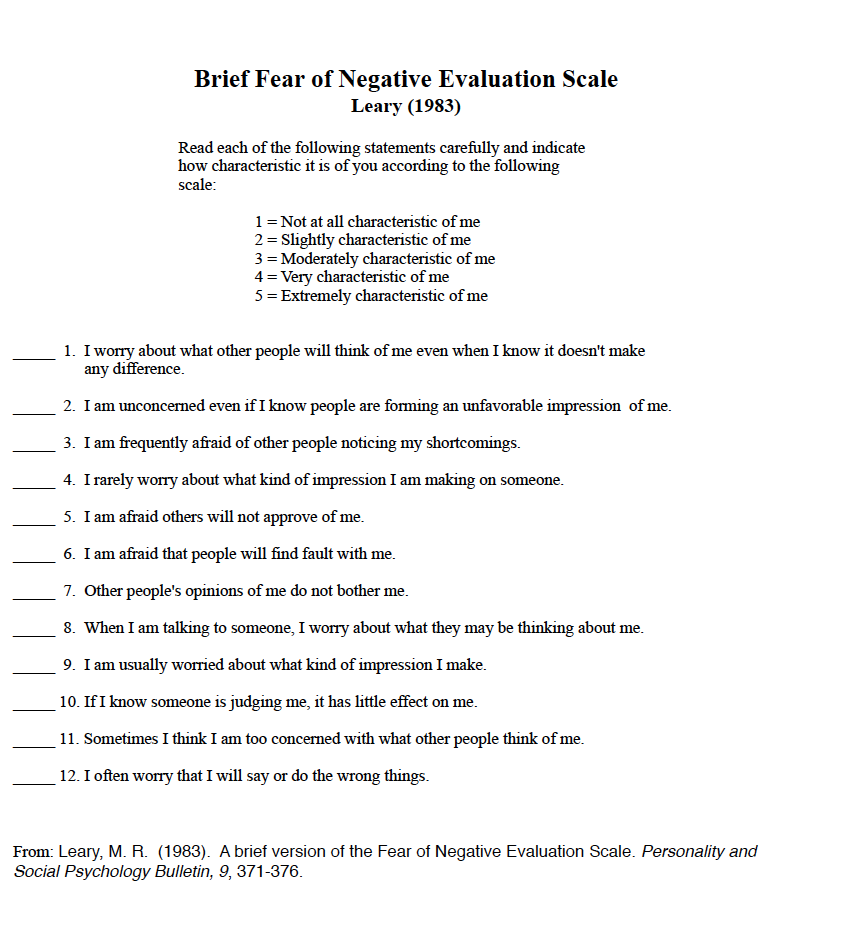
**

**Appendix G: Heterosexist, Harassment, Rejection, and Discrimination Scale (HHRDS)**

**
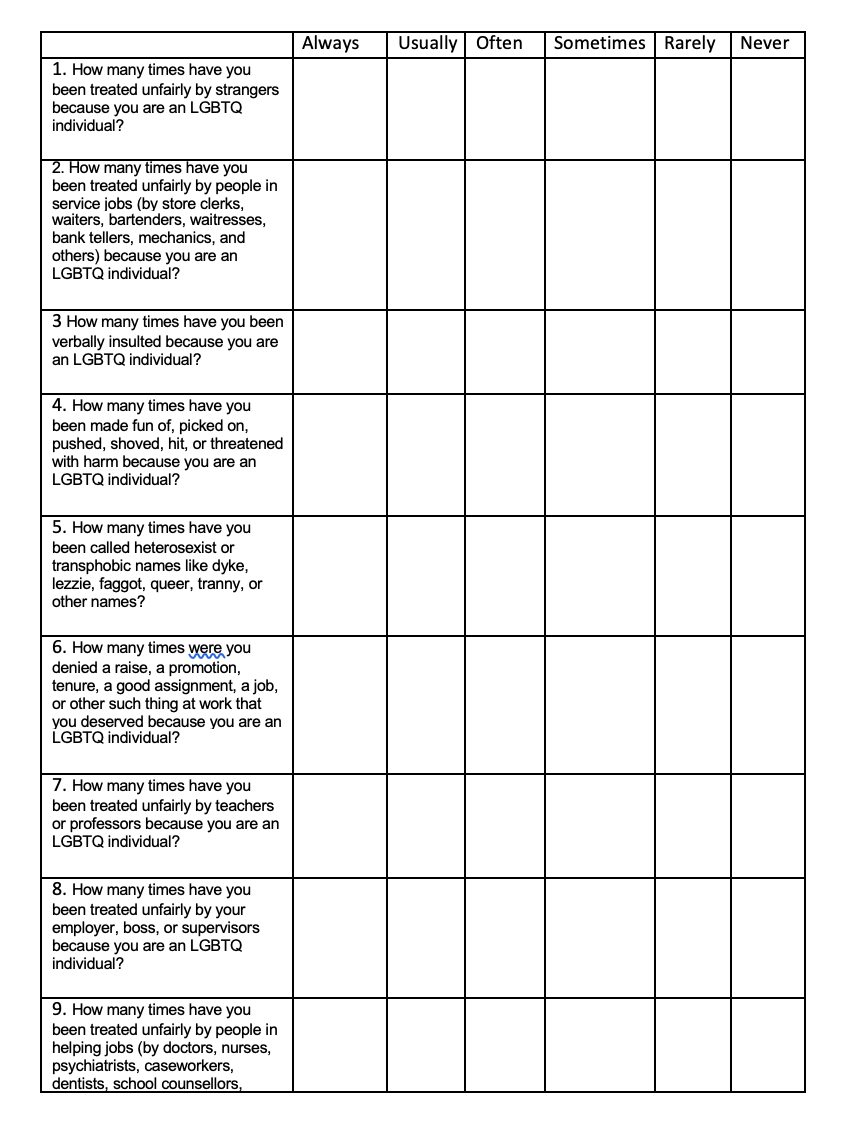
**

**
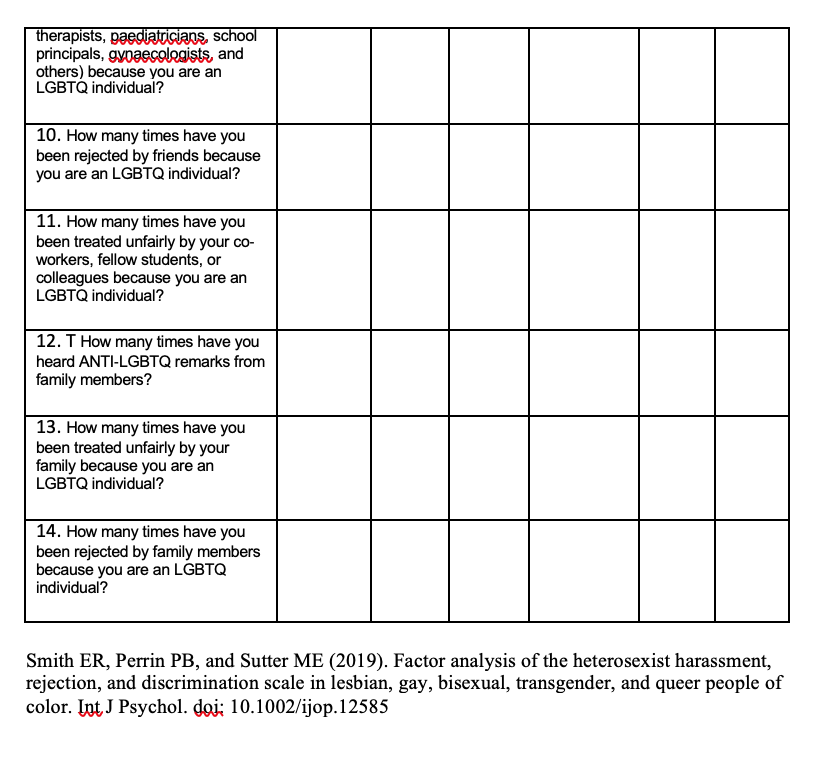
**

**Appendix H: Multidimensional Scale of Perceived Social Support (MSPSS)**

**
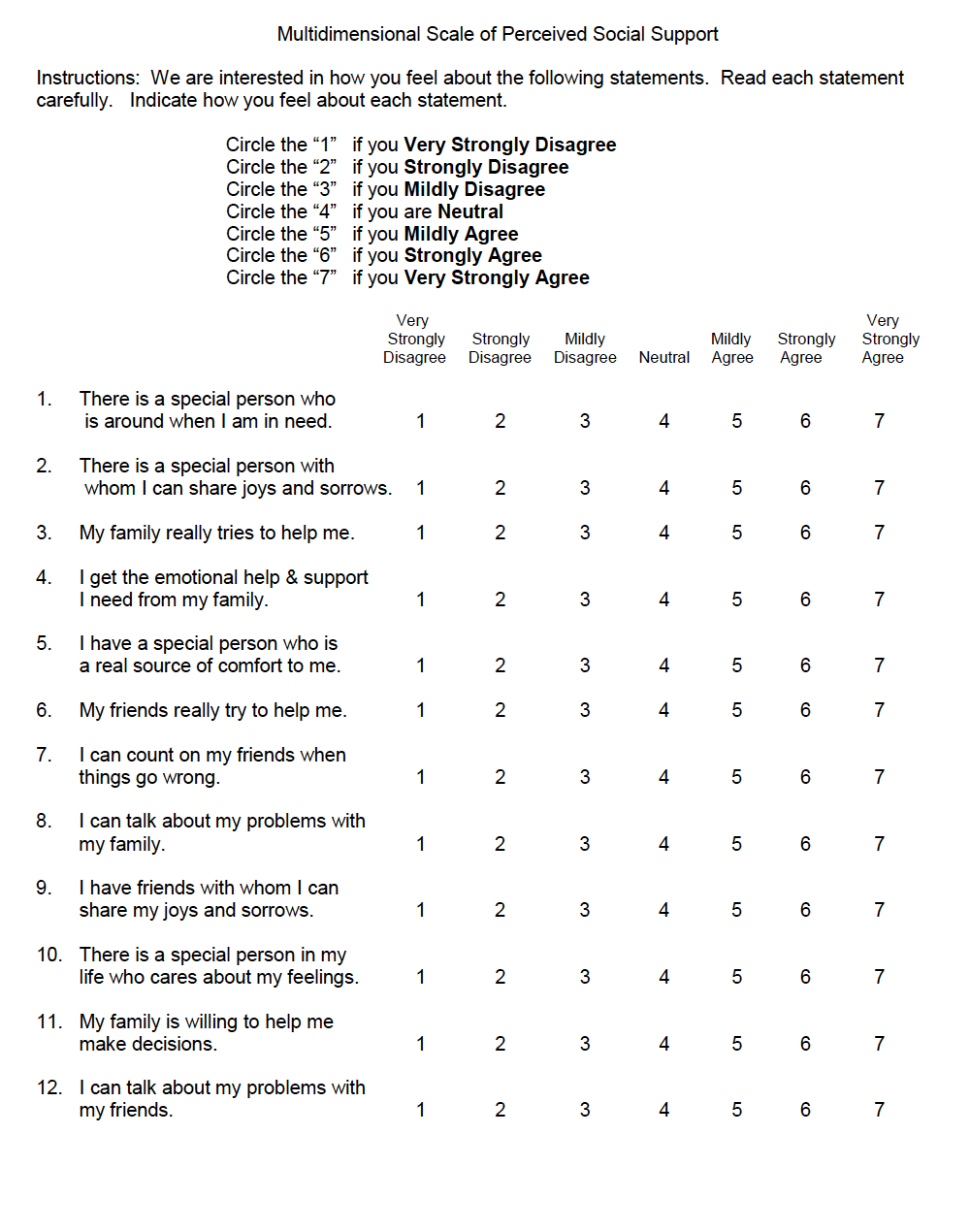
**From: Zimet GD, Dahlem NW, Zimet SG, Farley GK. The Multidimensional Scale of Perceived Social Support. Journal of Personality Assessment 1988;52:30-41.

**Appendix I: Your Guide to a Healthy Eating and Wellbeing**


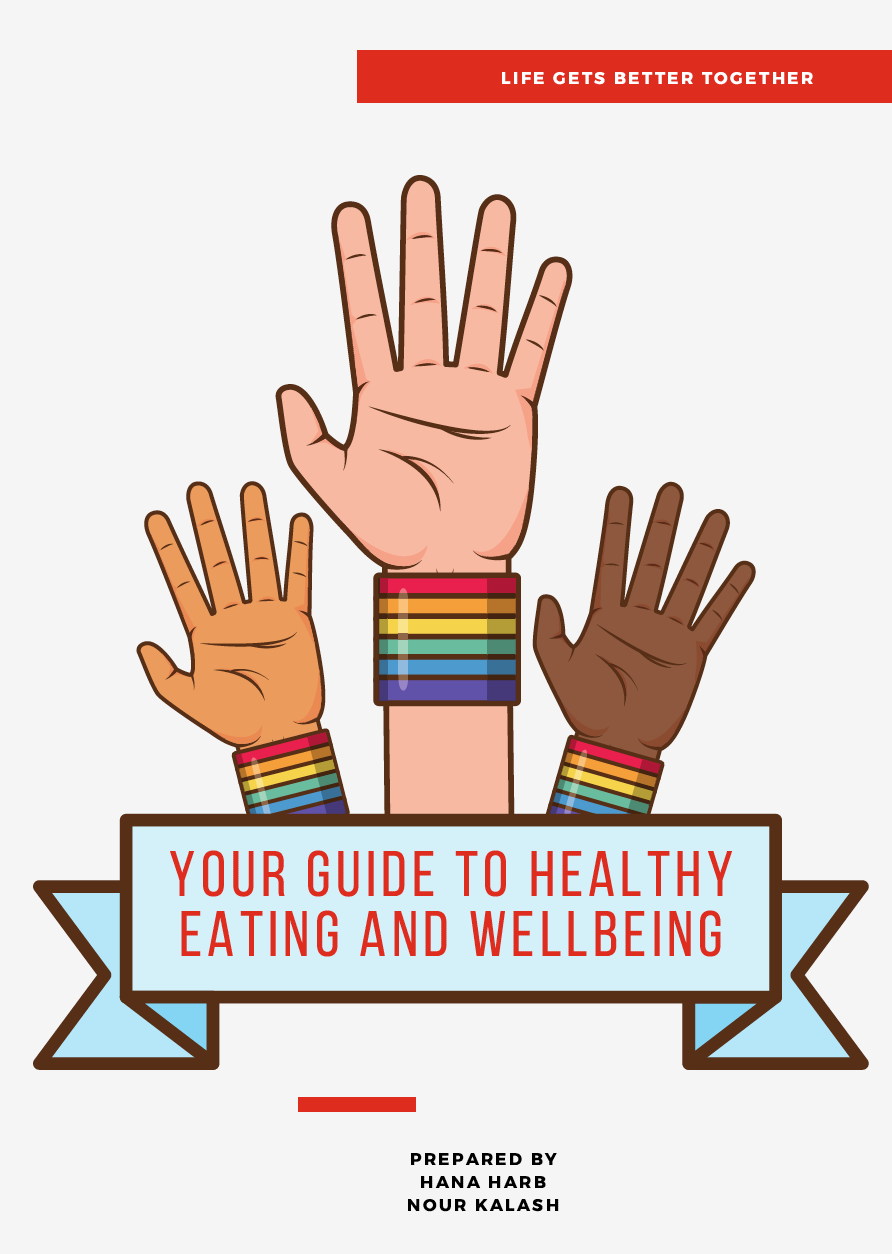


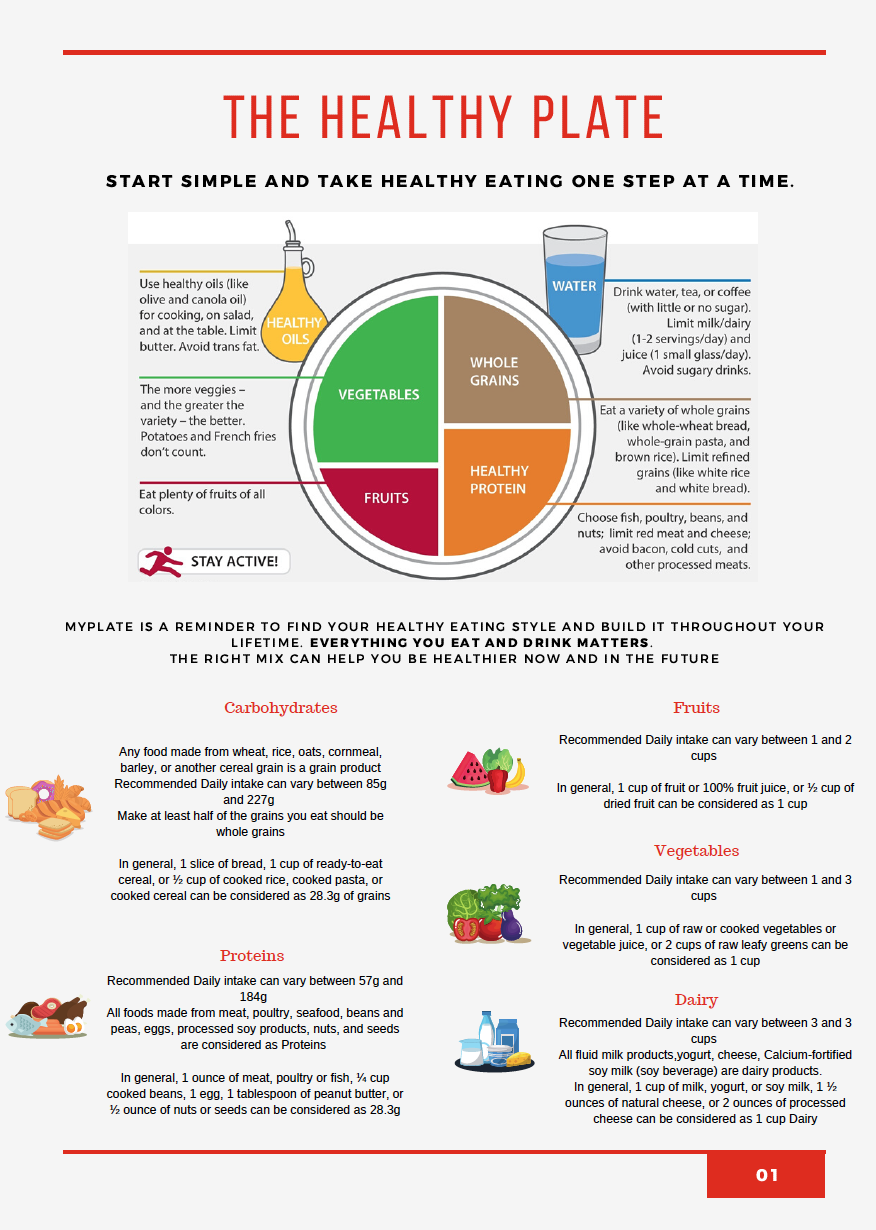


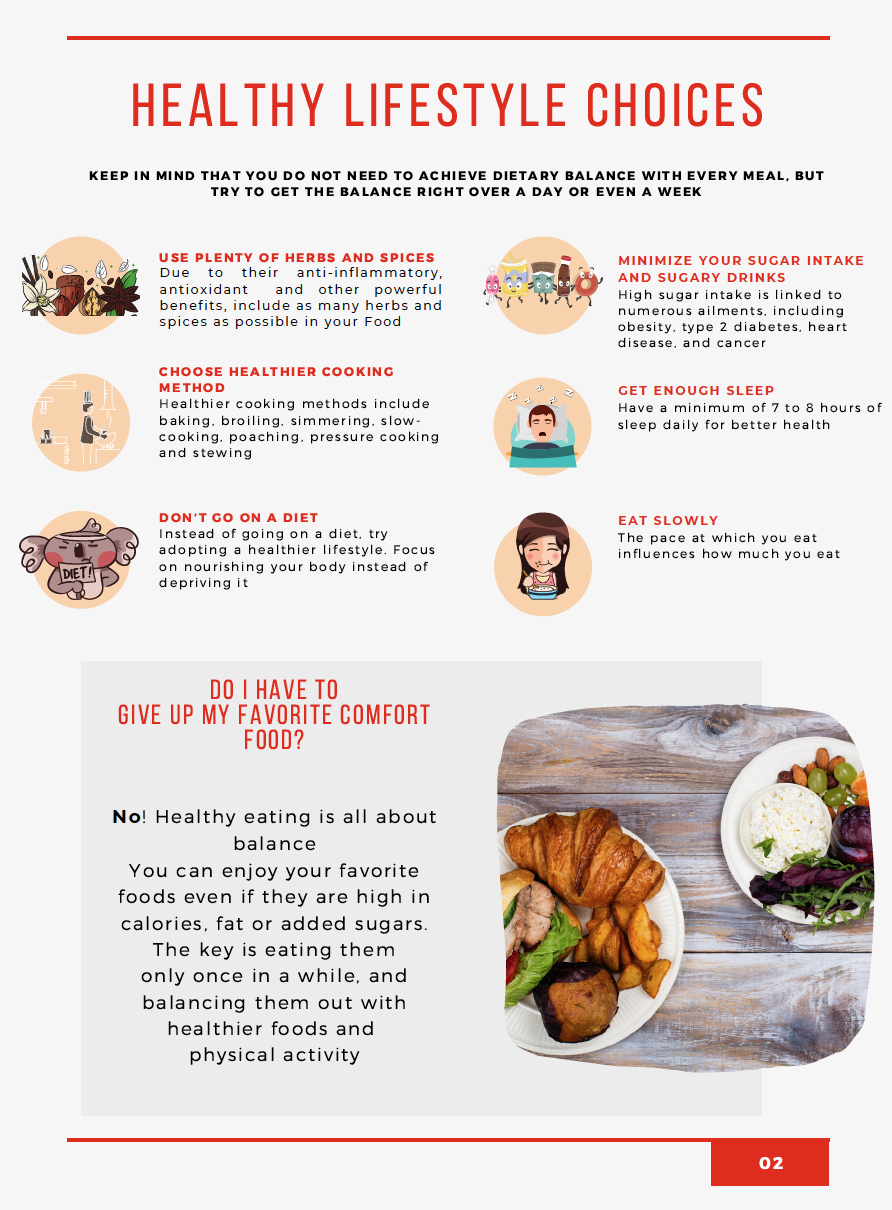


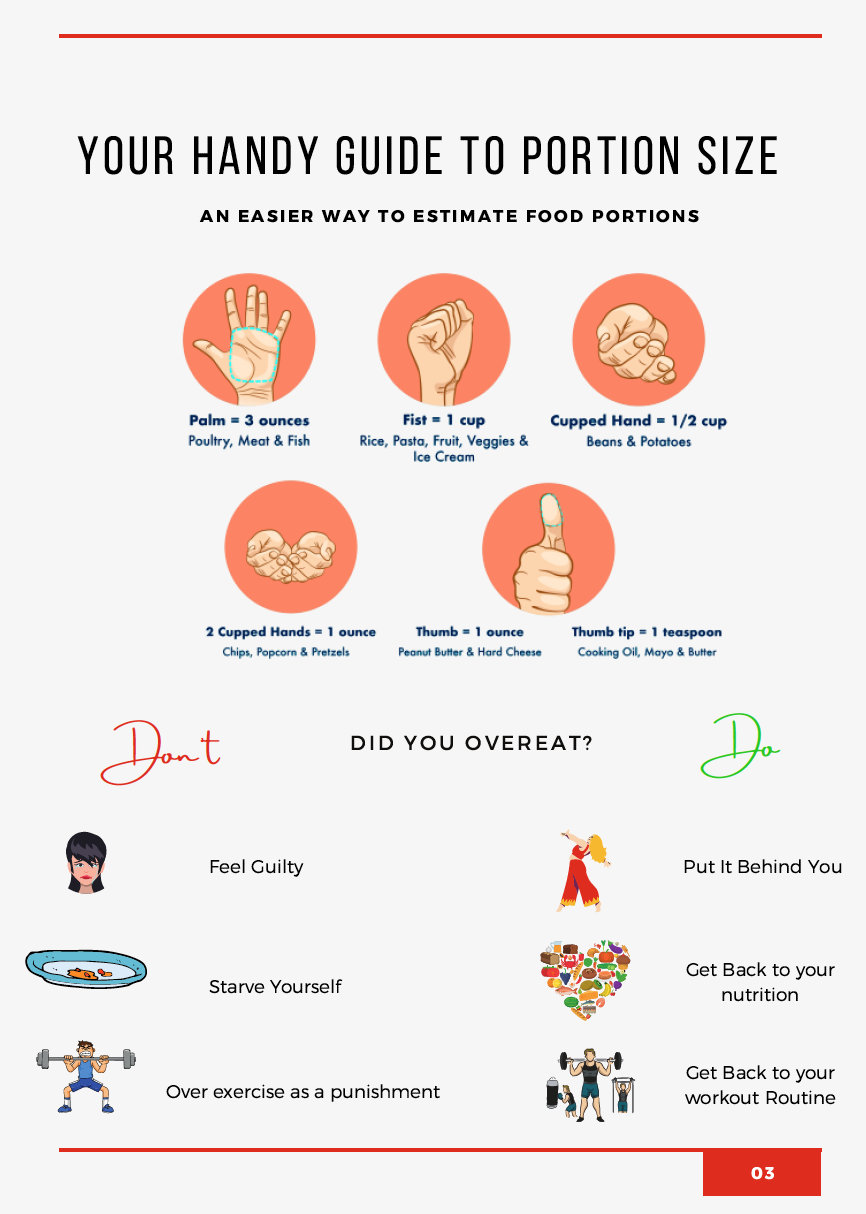


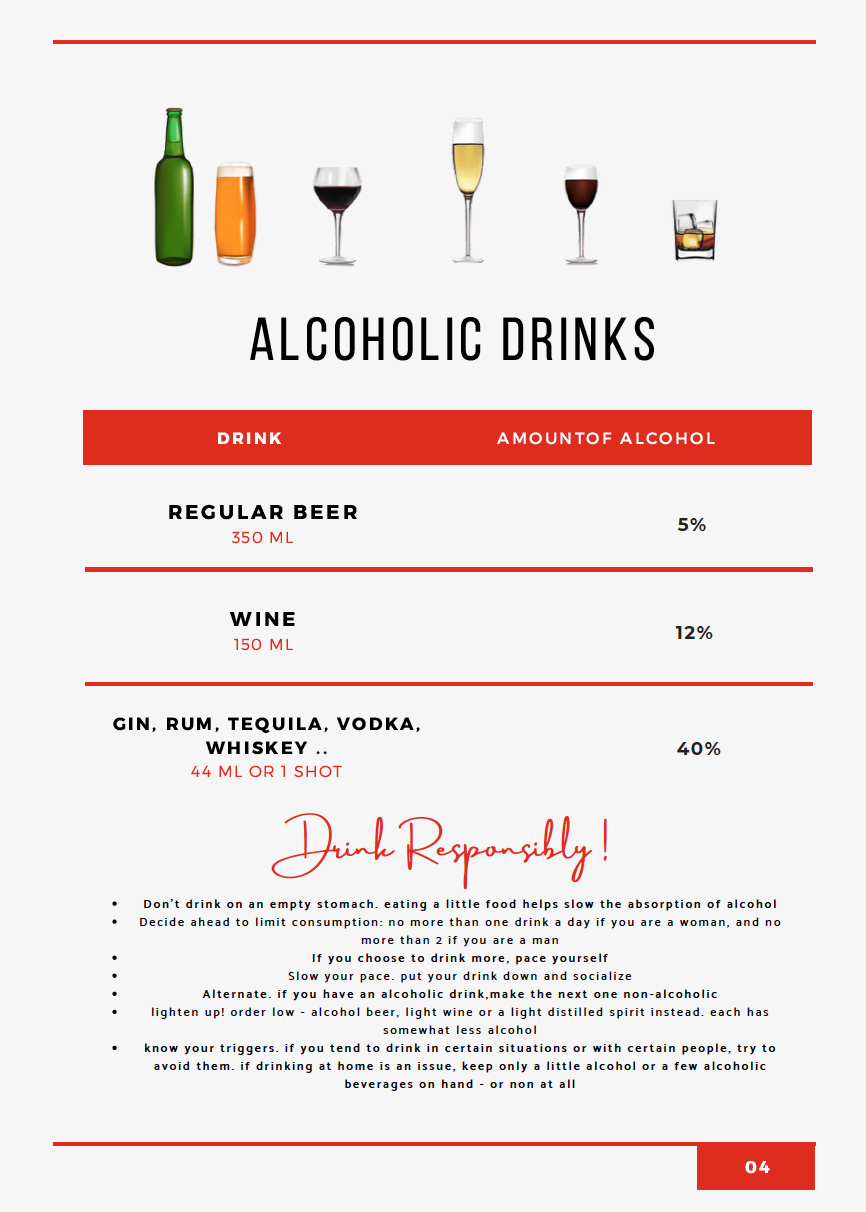


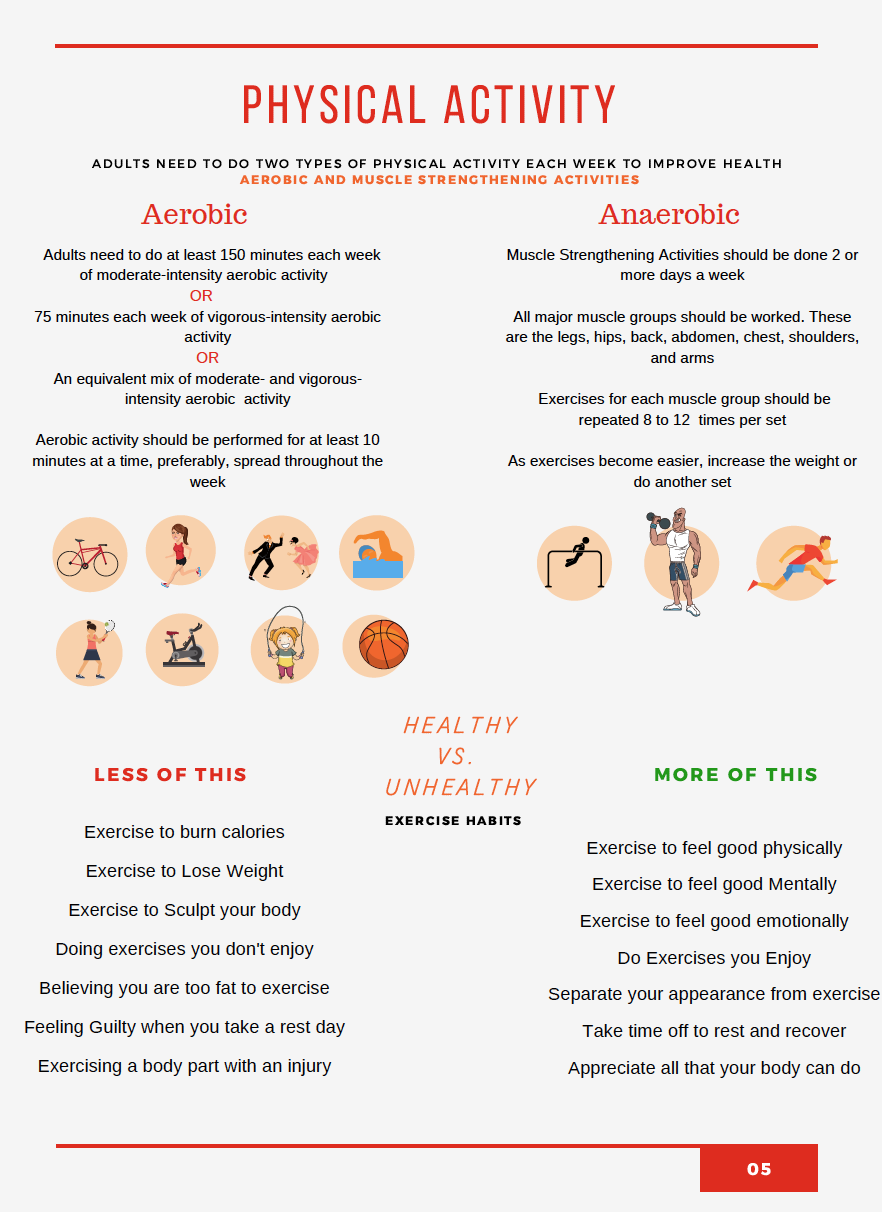


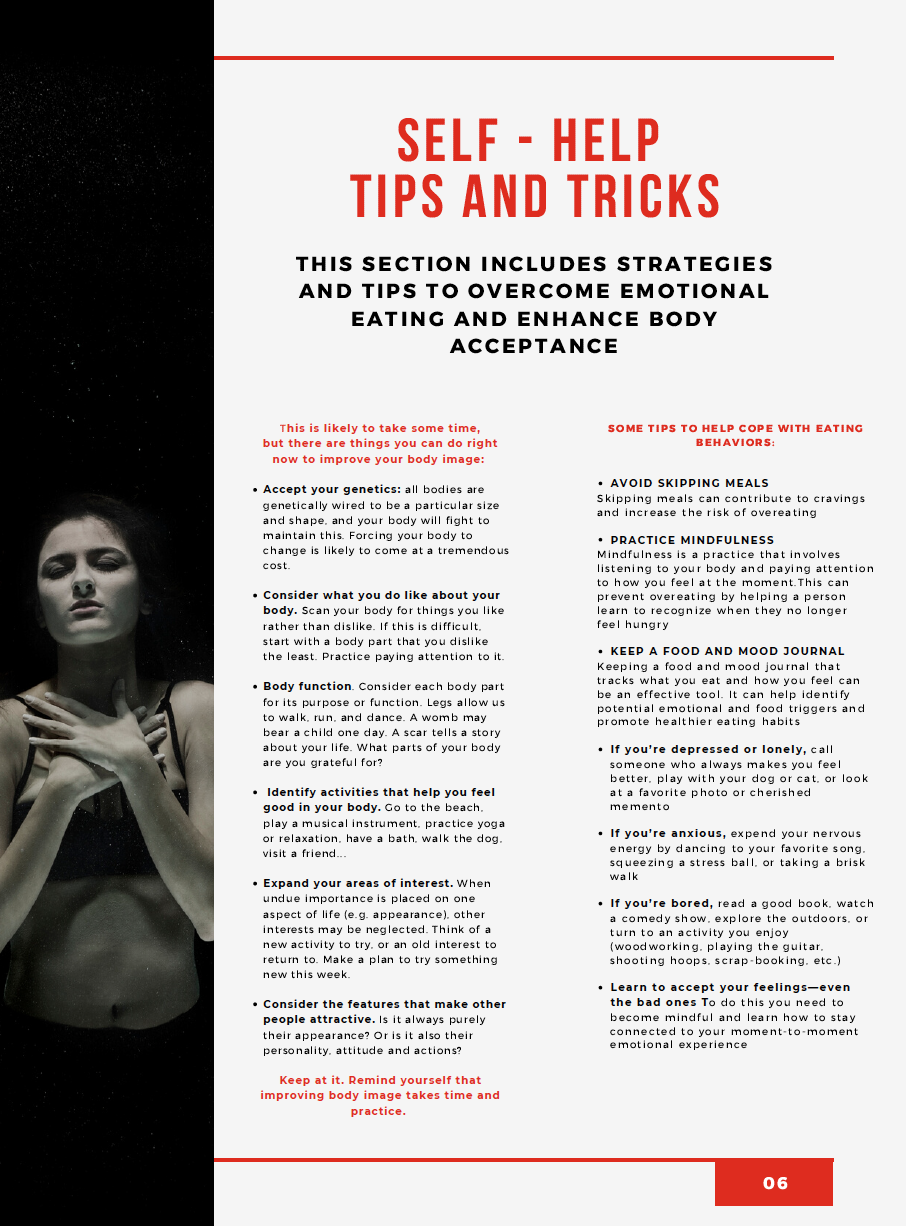


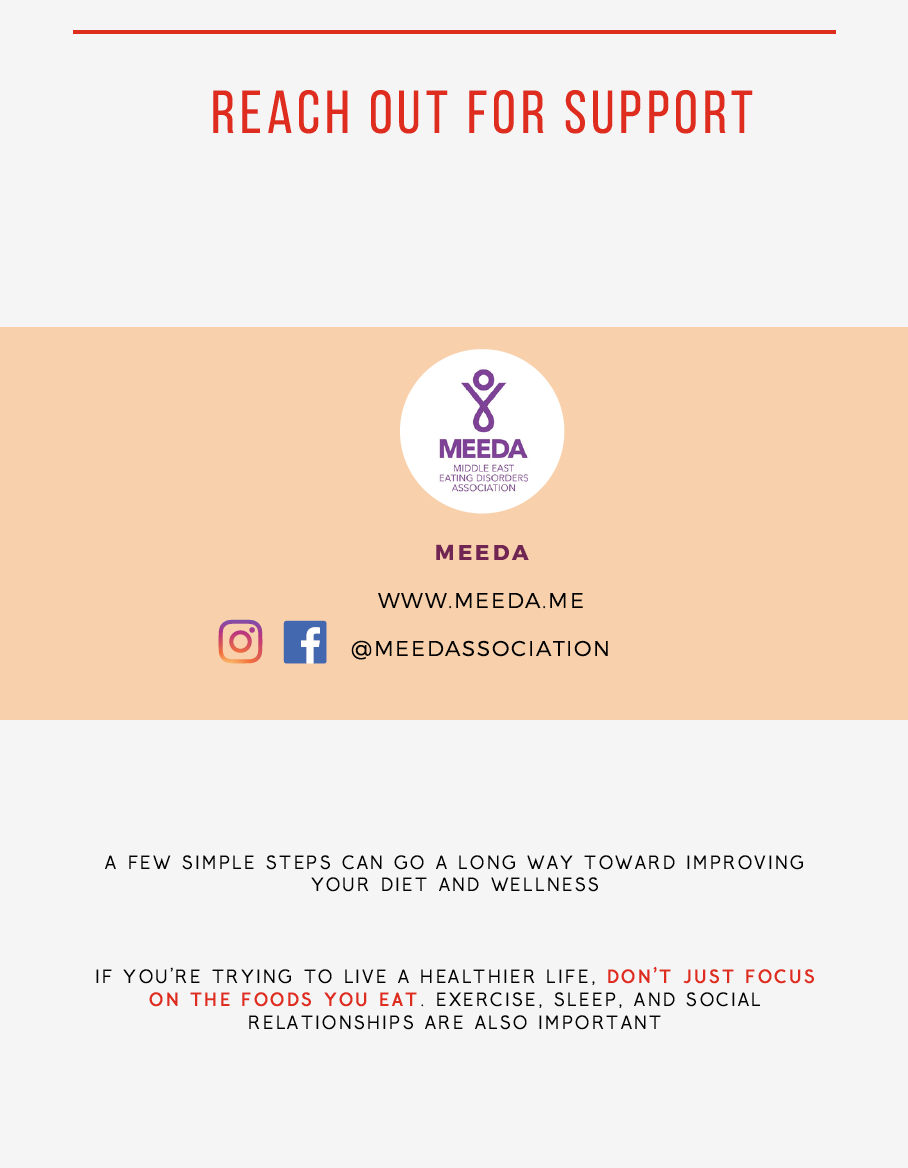


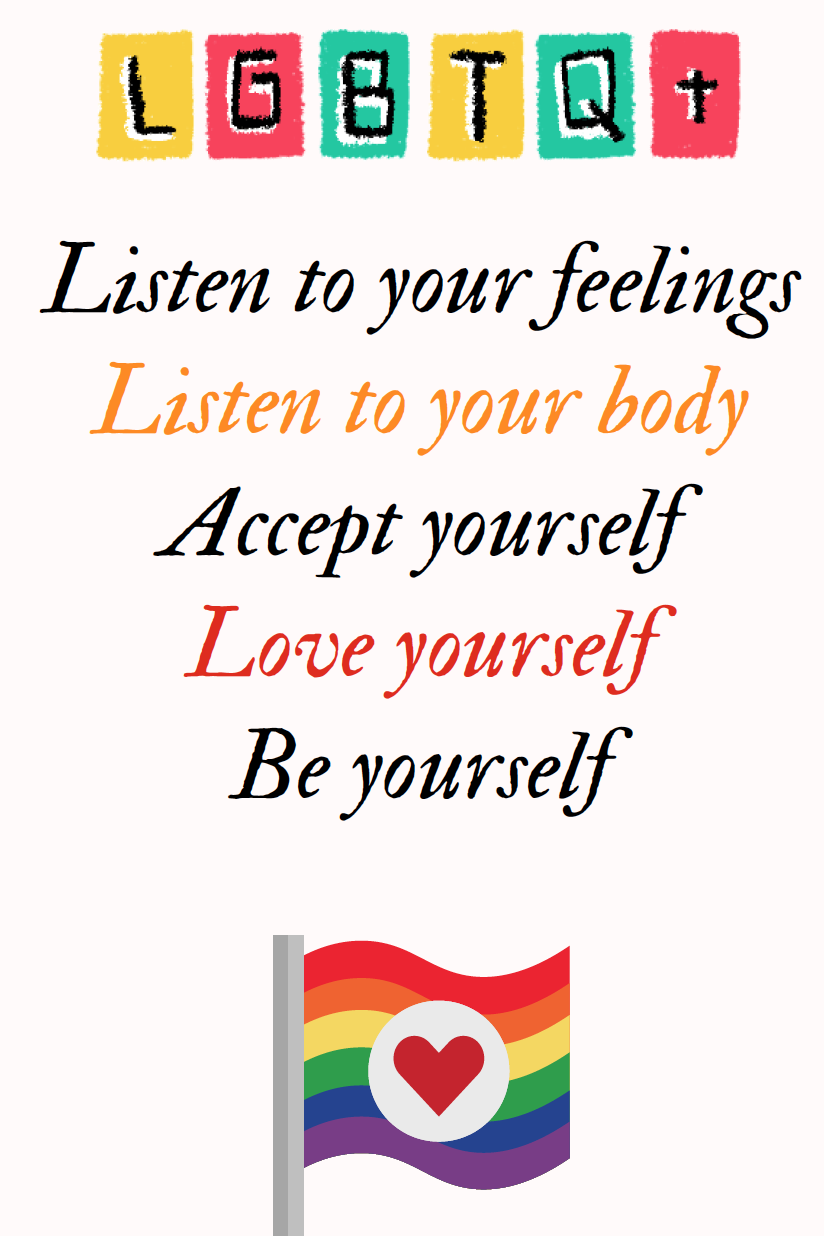

Supplement: Supplementary file 1 — Additional file 1: Appendix A. Informed consent. Appendix B. Sociodemographic, general health, and alcohol intake questionnaires. Appendix C. Eating disorder examination questionnaire (EDE-Q 6.0). Appendix D. Body appreciation scale (BAS-2). Appendix E. Generalized anxiety disorder (GAD-2). Appendix F. Brief fear of negative evaluation (BFNE). Appendix G. Heterosexist, harassment, rejection, and discrimination scale (HHRDS). Appendix H. Multidimensional scale of perceived social support (MSPSS). Appendix I. Your guide to a healthy eating and wellbeing. [file 40337_2023_810_MOESM1_ESM.docx]
